# Incidence of Enteric Fever in a Pediatric Cohort in North India: Comparison with Estimates from 20 Years Earlier

**DOI:** 10.1093/infdis/jiab046

**Published:** 2021-11-23

**Authors:** Bireshwar Sinha, Temsunaro Rongsen-Chandola, Nidhi Goyal, Alok Arya, Chandra Mohan Kumar, Aparna Chakravarty, Mohammed Aslam, Deepak More, SEFI tier 1 collaborators, Jacob John, Venkata Raghava Mohan, Ashish Bavdekar, Shanta Dutta, Gagandeep Kang

**Affiliations:** 1 Centre for Health Research and Development, Society for Applied Studies, New Delhi, India; 2 Department of Pediatrics, Hakeem Abdul Hameed Centenary Hospital, Hamdard Institute of Medical Sciences & Research, New Delhi, India; 3 Clinical and Research Laboratories, Society for Applied Studies, New Delhi, India

**Keywords:** Enteric fever, Typhoid, Children, Burden, Cohort, North India

## Abstract

**Background:**

An earlier cohort in 1995–1996 showed a very high burden of typhoid in Delhi. Our aim was to estimate the current overall and age-specific incidence of culture-confirmed enteric fever among children aged 6 months to 15 years in Delhi.

**Methods:**

We enrolled a cohort of 6000 children aged 6 months to <14 years in South Delhi and followed them up weekly for 24 months or until 15 completed years of child age, whichever was earlier. Blood culture to confirm enteric fever was done in children with ≥3 consecutive days of fever.

**Results:**

We recorded a total of 14 650 episodes of fever in the 11 510 person-years (PY) of follow-up. A total of 81 fever episodes were positive for enteric fever. The incidence (95% confidence interval) of all enteric fever was 703.7 (560.5–874.7) per 100 000 PY. The incidences of typhoid and paratyphoid fevers were 608.1 (95% confidence interval, 481.1–768.7) and 111.7 (59.5–191.1) per 100 000 PY, respectively, highest among children aged 10–15 years.

**Conclusions:**

Despite a 35% reduction in incidence compared with the 1995–1996 cohort, our study suggested a substantial burden of enteric fever in the population. Continued efforts to improve water, sanitation, and hygiene parameters along with implementation of novel vaccination strategies and disease surveillance can help achieve the goal of disease elimination.

India is endemic to typhoid and paratyphoid fever. The overall pooled incidence of culture-confirmed typhoid in India is estimated to be 377 per 100 000 person-years (PY), based on 3 community-based studies conducted between 1995 and 2006, with most cases among children aged 2–4 years [1]. There was high heterogeneity in the burden, ranging from 215 per 100 000 PY in Kolkata to 980 per 100 000 PY in New Delhi [1–4]. In the 1995–1996 New Delhi cohort, the incidences among participants <20 years of age and children <5 years old were 1590 and 2730 per 100 000 PY, respectively; the highest burden was among 2–3-year-old children, at 5160 per 100 000 PY [2]. Based on these findings, typhoid polysaccharide vaccine was introduced in Delhi in November 2004 as a single dose for children 2–5 years of age [5, 6]. The decision on vaccine introduction in other parts of the country could not reach a consensus due to lack of sufficient evidence on disease burden.

Currently, typhoid conjugate vaccines (TCVs) are available, which are more efficacious with persistently higher geometric mean titers of immunoglobulin (Ig) G Vi antibody 3–5 years after vaccination compared with the polysaccharide vaccine [7]. The World Health Organization (WHO) recommends routine use of TCVs in children >6 months of age in typhoid-endemic countries, prioritizing countries with a high burden or with antibiotic-resistant typhoid [8]. To determine the target population for TCV administration and delivery strategy, there is a need to look again at the local epidemiology of typhoid fever, including antimicrobial resistance patterns. There is a perceived decline in the incidence and severity of typhoid fever based on hospital-based reports [9, 10]. This decline might possibly be due to improvements in water, sanitation, and hygiene (WASH) indicators, sociodemographic parameters, and/or local vaccine introduction. The decline may also be apparent owing to underreporting and delayed healthcare seeking, poor access to quality diagnostic facilities, and indiscriminate early antibiotic use in febrile illness, which affects culture positivity and increases antimicrobial resistance. This needed further investigation.

Our aim was to estimate the overall and age-specific incidence of culture-confirmed enteric fever (typhoid and paratyphoid) among children 6 months to 15 years old in New Delhi. Our strategy was to investigate febrile episodes through active weekly surveillance over a 2-year period. We also report treatment practices associated with acute febrile illness in the community, typhoid vaccine use, sociodemographic factors, and food and WASH practices, including drinking water quality, to explain any observed differences in disease burden over time in comparison with the 1995–1996 New Delhi cohort [2].

## METHODS

### Study Population

We are part of a multisite cohort study on Surveillance of Enteric Fever in India (SEFI) among children; the common protocol with sample size calculation is published elsewhere [9]. Here we describe the site-specific methods. The study was conducted in 2 contiguous blocks of Sangam Vihar, a low-income urban resettlement neighborhood in the south district of New Delhi.

As a first step, a door-to-door survey was conducted in the 2 blocks to line list the households with potentially eligible children. Families were screened using an interview process to determine their likelihood of moving out of the study area and/or adhering to study procedures, especially blood specimen collection during fever. Enlisted eligible children who were aged 6 months to <14 years and were likely to stay in the study area for 2 years were enrolled in the cohort from contiguous households until a sample size of 6000 was attained. Before screening, written informed consent was obtained from the parents; verbal assent was obtained from children aged 7–12 years and written assent from those >12 years old. All enrolled children from a household were followed up for 24 months or until 15 years of age, whichever was earlier. Institutional ethics approval was obtained. The study was registered prospectively in the Clinical Trial Registry of India (CTRI/2017/09/009719).

### Acute Febrile Illness Surveillance

We conducted active weekly contacts either by phone or by home visits, with ≥1 face-to-face contact every 4 weeks to collect information on fever, illness, or hospitalizations. In addition, a monthly mobile recharge of 50 Indian rupees was provided to all enrolled families to encourage early reporting of fever to the study team, by telephone. Quality checks were done by the study supervisor if contact with primary caregiver was not made for 2 consecutive weeks.

Fever was defined as a caregiver-reported or recorded temperature of ≥38ºC (100.4ºF). When fever was reported, our study team visited the participant’s home as early as possible. Families were provided with a digital thermometer and fever diary card, were trained to document temperature 3 times in a day, and were advised to avoid over-the-counter medications. The team made daily contact until resolution of fever, defined as 3 consecutive fever-free days. If fever lasted for ≥3 days, the child was referred to the study fever clinic at Hakeem Abdul Hameed Centenary Hospital; referral was facilitated whenever possible.

Blood cultures were performed to confirm enteric fever in children with ≥3 consecutive days of fever. Culture was deferred if the child was afebrile in the preceding 12 hours or if clinical suspicion was low; however, if fever reappeared in the next 24 hours, a blood culture was performed. Blood culture was withheld if one had been conducted within the previous 2 weeks in the same child, unless repeated culture was advised by the treating physician. Prior antimicrobial therapy was not a contraindication for blood culture. Children with culture-confirmed enteric fever were managed at the study fever clinic or any other preferred place of care seeking. Medical expenses related to culture-confirmed enteric fever were covered, and hospitalizations were facilitated at the Hakeem Abdul Hameed Centenary Hospital. If the participant visited other health facilities for care seeking, information related to the fever episode was collected during the weekly contacts.

### Surveillance for WASH and Food Practices

Information was collected regarding sociodemographic details, WASH practices, and consumption of street food in study households during the months of April to September in 2018 and 2019. Given that all eligible children from a household were enrolled, to best capture the eating-out practices among children in a household, we recorded this information for the eldest enrolled child. To determine the prevalence of safe drinking water among households in the area in terms of microbial quality (*Escherichia coli* or thermotolerant coliform bacterial counts in a 100-mL drinking water sample), a subsurvey was conducted in December 2019 in 108 households selected by stratified random sampling. The sample size for this exercise was calculated assuming that approximately 50% households in the community would have access to microbially safe drinking water with an allowable error of 20%. The study area was divided into 12 contiguous areas (strata) of similar sizes. Nine households were selected from each of these 12 areas, using computer-generated random numbers. Drinking water samples (1.5 L) were collected from the place of drinking water storage in these households to test for coliform count, pH, and total dissolved solids.

### Laboratory Methods

For blood culture in children aged 6 months to 1 year or >1 year, 3 or 5 mL of blood, respectively, was inoculated into the BACTEC Peds Plus bottle by a trained laboratory personnel. Specimens were transferred to the Clinical and Research Laboratories, Society for Applied Studies (CRL SAS) within 4 hours after specimen collection, at ambient temperature. Blood culture was done using BACTEC FX 40 (Becton Dickinson) at CRL SAS. Bottles with positive signal for growth were processed for identification of the organism, using Gram stain (K001; Hi-Media Laboratories), subculture, and biochemical testing. Serotype identification was done by slide agglutination method using antiserum from CRI. Antimicrobial susceptibility was analyzed with the Kirby-Bauer disc diffusion method for all enteric fever isolates, which were archived for future genomic characterization. The CRL SAS laboratory participated in external quality assurance system coordinated by the central SEFI study team for the laboratory processes. Drinking water quality assessments, including coliform count (performed using the membrane filtration method) was conducted at TUV SUD South Asia (https://www.tuvsud.com/en-in).

### Data Management

Data were collected on tablets using that android application package EntericFev, developed in house by the SEFI team and stored in a secured Amazon cloud-based server. Data were monitored using a dashboard-based system; weekly reports were generated and reviewed. A comprehensive audit trail was maintained. Quality assurance was performed by a central independent team of experts.

### Statistical Analysis

Statistical analysis was done using Stata 16.0 MP software (StataCorp). Person-years for each child were calculated from the date of enrollment to censorship (end of study, withdrawal, death, or no face-to-face contact for ≥90 days). During follow-up, if a participant could not be contacted for ≥2 consecutive weeks and subsequently became available, the information of only the previous 2 weeks from the date of subsequent contact was captured and considered in person-years of follow-up, and the intervening time period was not considered in the analysis. The total person-years of follow up in the study were divided into 3 age categories: 6 months to <5 years, 5 to <10 years, and 10–15 years, determined by chronological age. The age-specific incidence rates of culture-confirmed enteric fever (typhoid and paratyphoid) or acute febrile illness were calculated as the number of new events in the specific age interval divided by total person-years of follow-up contributed by all children at risk in this interval, allowing for children to move to higher age categories. Confidence intervals (CIs) were calculated using the Poisson option in Stata.

## RESULTS

**Figure 1.**
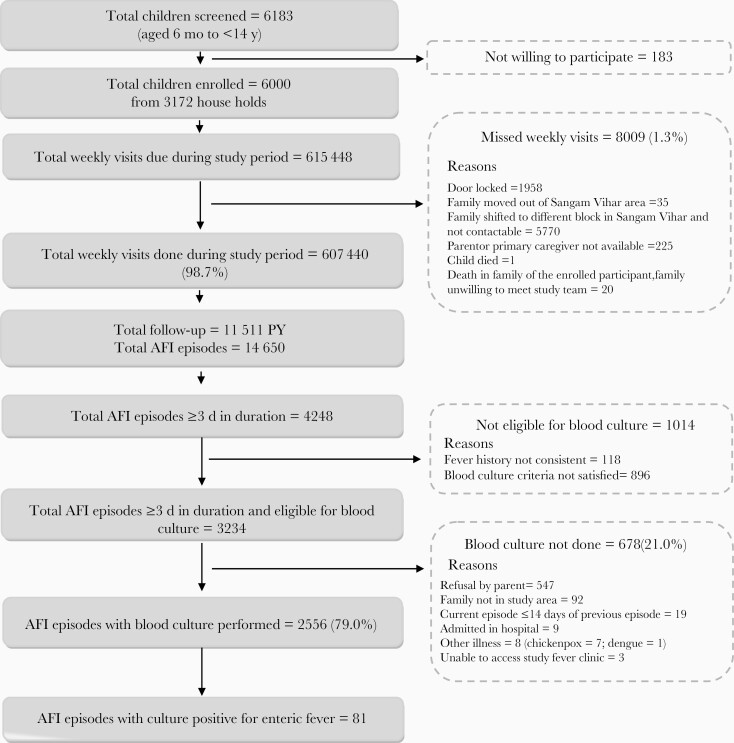
Study flow. Abbreviation: AFI: acute febrile episode; PY: person-years.

We followed up a fixed cohort of 6000 children from 3172 households from the date of first enrollment, 23 October 2017, to the date of completed follow-up, 26 February 2020. Of the total scheduled weekly visits, 1.3% (8009 of 615 448) were missed. In this cohort we had a total of 11 511 PY of follow-up, of which 2871, 4790, and 3850 PY, respectively, were contributed by children aged 6 months to <5 years, 5 to <10, and 10–15 years (Figure 1). During this follow-up period a total of 4 child deaths (2 of pyrexia of unknown origin, 1 of thalassemia, and 1 of leukemia) and 151 hospitalizations were reported.

### Characteristics of the Study Population

About two-thirds (73%) of the children enrolled belonged to nuclear families with median monthly income of 143 USD; 68% of these households were overcrowded (Table 1). In this cohort, 14% of the children (820 of 6000) had received a typhoid vaccine previously, a polysaccharide vaccine in 95% of them (778 of 820). The mean (standard deviation) age at typhoid vaccination was 30.2 (9.9) months. Piped water from the Delhi Municipal Corporation (83%) and bottled water (10%) were the major sources of household drinking water. In the subsurvey, we found that 14% households (16 of 108) had coliforms or *E. coli* in their drinking water; the median level of total dissolved solids was 130 mg/L (interquartile range [IQR], 65–151), and the mean (standard deviation) pH was 7.2 (0.5). Almost all used sanitary toilets, of which one-third has flush systems; 14% of the households shared toilet with other families. The practice of consuming ready-to-eat food and ice cream from street vendors at least once a week was noted in 53% and 36% households, respectively (Table 1).

**Table 1.** Individual and Family Characteristics of Study Population

	No. With Characteristic/Total No. (%)^a^
Baseline Individual and Family Characteristics	
Female sex	2933/6000 (48.9)
Age at enrollment	
6 mo to <5 y	1926/6000 (32.1)
5 to <10 y	2547/6000 (42.5)
10 to <14 y	1527/6000 (25.5)
Receipt of any typhoid vaccine	820/6000 (13.7)
Type of typhoid vaccine received	
Polysaccharide	778/820 (94.9)
Conjugate	19/820 (2.3)
Unclear	23/820 (2.8)
Place of vaccination	
Government facility	782/820 (95.4)
Private facility	38/820 (4.6)
Sociodemographic parameters	
Type of family	
Nuclear	2268/3123 (72.6)
Three generation	171/3123 (5.5)
Joint	684/3123 (21.9)
Family size, mean (SD), no.	5.7 (2.2)
Highest educational level in family, mean (SD), y of schooling	10.9 (3.3)
Type of house^b^	
Pucca	3101/3123 (99.3)
Mixed	17/3123 (0.5)
Kutcha	5/3123 (0.2)
Overcrowding present (>2.5 persons per living room)	2133/3123 (68.3)
Separate kitchen available	2129/3123 (68.2)
Primary fuel used for cooking in household	
Liquefied petroleum gas	3115/3123 (99.7)
Other	8/3123 (0.3)
Monthly income, median (IQR), $ ^c^	143 (114–214)
WASH parameters	
Source of drinking water	
Piped water in household	2585/3123 (82.8)
Bottled water	323/3123 (10.3)
Public tap/standpipe	90/3123 (2.8)
Tube well	39/3123 (1.3)
Tanker truck	84/3123 (2.7)
Other	2/3123 (0.06)
Practice of water treatment before drinking^d^	1137/3123 (36.4)
Microbiological quality of drinking water^e^	
Presence of any coliforms/*Escherichia coli* per 100 mL	16/108 (14.8)
Presence of >100 coliforms/*E. coli* per 100 mL	15/108 (13.9)
Presence of 10–100 coliforms/*E. coli* per 100 mL	1/108 (0.9)
Total dissolved solids in household drinking water, median (IQR), mg/L^d^	130 (65–151)
Households with total dissolved solids >600 mg/L in drinking water^d^	3/108 (2.7)
pH of household drinking water, mean (SD)^e^	7.2 (0.5)
Type of toilet facility used	
Flush toilet	1028/3123 (32.9)
Pit latrine	2088/3123 (66.9)
Ventilated improved pit latrine	7/3123 (0.2)
Sharing of toilet with other households	421/3123 (13.5)
Food practices^f^	
Consumption of ready-to-eat-food from street vendors	
≥1 time/wk	1353/3123 (52.9)
≤1 time/2 wk	1190/3123 (38.1)
Never	280/3123 (9.0)
Consumption of ice cream from street vendors	
≥1 time/wk	1110/3123 (35.5)
≤1 time/2 wk	1200/3123 (38.4)
Never	813/3123 (26.1)

Abbreviations: IQR, interquartile range; SD, standard deviation; WASH, water, sanitation, and hygiene.

^a^Data represent no. of children (or families) with characteristic/total no. (%) unless otherwise specified. In this cohort of 6000 children from 3172 households, the survey on demographic characteristics and WASH and food practices was completed in 3123 households with 5916 children; the denominators represent these totals.

^b^A pucca house is one that has walls and roof made of bricks, stones packed with cement. Kutcha houses are made of material other than those mentioned above, including bamboos, mud, grass, reeds, and thatch.

^c^Note: $1 (US) is equivalent to 70 Indian rupees.

^d^Methods of water treatment include boiling, chlorination, and filtration.

^e^Drinking water quality was tested in 108 randomly selected households.

^f^Information on food practices was collected for the eldest enrolled child from each household when multiple children were enrolled from the same household.

### Acute Febrile Illness and Enteric Fever Surveillance

**Figure 2.**
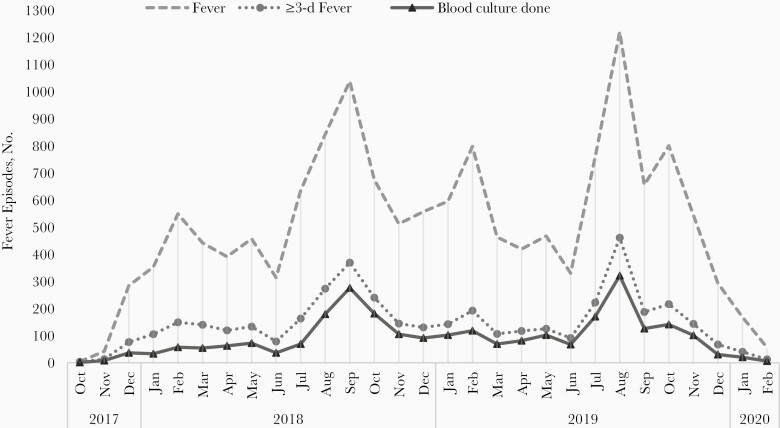
Fever episodes and blood cultures during the study period, by month.

Overall, there were 14 650 episodes of fever with peaks during the months of June to November (Table 2 and Figure 2). The overall incidence of fever was 126/100 PY (95% CI, 124.0–128.1), with the highest incidence in children <5 years old 197.6/100 PY (192.5–202.8). The median (IQR) duration of a fever episode was 2 (1–4) days. In 38% of the fever episodes (5529 of 14 650), a known antibiotic was prescribed and was initiated around day 2 (IQR, 1–4) of fever onset. Oral β-lactams, cephalosporin, and macrolides were the most commonly prescribed antibiotics. Of the 4248 fever episodes lasting ≥3 days, 76% (3234 of 4248) were eligible for blood culture examination. Blood culture was conducted in 79% (2556 of 3234) of these eligible episodes (Figure 1). The median (IQR) time of performing blood culture was day 4 (4–5) of fever.

**Table 2.** Incidence of Fever Episodes by Age Category

Age Category	Follow-up Time, PY	All Fever Episodes		Fever Episodes Lasting ≥3 d	
		Total No.	Incidence (95% CI), No./100 PY	Total No.	Incidence (95% CI), No./100 PY
6 m to <5 y	2870.8	5669	197.5 (192.4–202.7)	1606	55.9 (53.3–58.7)
5 y to <10 y	4790.2	5513	115.1 (112.1–118.2)	1655	34.6 (32.9–36.3)
10 y to 15 y	3849.6	3468	90.1 (87.1–93.1)	987	25.6 (24.1–27.3)
All children	11 510.5	14 650	152.6 (125.2–129.4)	4248	36.9 (35.8–38.0)

Abbreviations: CI, confidence interval; PY, person-years.

**Figure 3.**
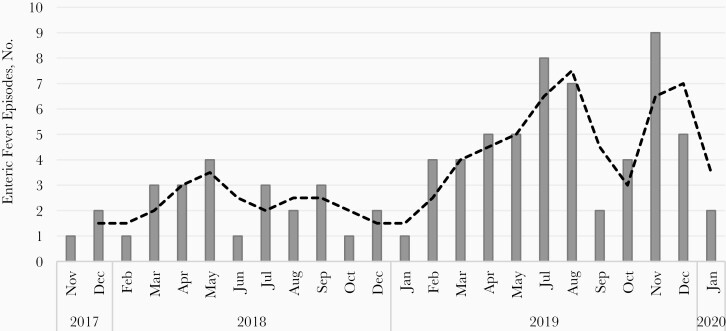
Culture-confirmed enteric fever episodes during the study period, by month.

A total of 81 fever episodes were positive for enteric fever based on blood culture; 70 of 81 episodes were confirmed with *Salmonella* Typhi, and 13 of 81 episodes were confirmed with *S.* Paratyphi A. Cases of coinfection with *S.* Typhi and *Salmonella* Paratyphi were observed in 2 participants aged 8 and 4 years [11]. No case of reinfection in a child was observed. However, a case of typhoid fever followed by paratyphoid fever was observed in a child at ages 9.3 years and 10.4 years, respectively. The blood culture positivity rate in our cohort was 3.2% (95% CI, 2.5%–3.9%). We observed more enteric fever episodes during May to December (monsoon and postmonsoon seasons), and were enteric fever episodes in 2019 than in 2018 (Figure 3).

The incidence of enteric fever was 704 (95% CI, 566–875) per 100 000 PY overall and 608 (481–769) and 113 (66–195) per 100 000 PY, respectively, for typhoid and paratyphoid fever. The incidence of both typhoid and paratyphoid fever was highest among children aged 10–15 years (Table 3). In the enteric fever cases, abdominal pain (74%) and nausea and vomiting (33%) were among the most common symptoms, other than fever (Table 4). The mean durations of typhoid and paratyphoid fever episodes were 10.2 and 7.7 days, respectively, and the mean duration of coinfections was 15.5 days. Of all the typhoid fever episodes, 17% (12 of 70) involved hospitalization, and the mean duration of hospitalization was 6.9 (2.8) days. Among children with typhoid fever, those hospitalized had higher temperature than those not hospitalized (mean highest daily temperature, 39.4 C vs 38.8 C, respectively). No antimicrobial resistance was found for ampicillin, azithromycin, or ceftriaxone in any of the *S.* Typhi or *S.* Paratyphi isolates (Supplementary Table 1). All the children recovered without any enteric fever–related complications, except in a 4-year-old girl with typhoid and paratyphoid coinfection who had hemodynamic shock and recovered after 11 days of hospitalization. No enteric fever–associated deaths were observed (Table 4).

**Table 3.** Incidence of Enteric Fever Episodes by Age Category

Age Category	Enteric Fever Episodes					
	All Enteric Fever^a^		Typhoid Fever		Paratyphoid Fever	
	Total No.	Incidence (95% CI), No./100 000 PY	Total No.	Incidence (95% CI), No./100 000 PY	Total No.	Incidence (95% CI), No./100 000 PY
6 mo to <5 y	16	557.3 (341.4–909.7)	14^b^	487.7 (288.8–823.4)	3^b^	104.5 (33.7–324.0)
5 to <10 y	31	647.2 (455.1–920.2)	30^b^	626.3 (437.9–895.7)	2^b^	41.8 (10.4–166.9)
10–15 y	34	883.2 (631.1–1236.1)	26	675.4 (459.9 –992.0)	8	207.8 (103.9–415.6)
All children	81	703.7 (566.0–874.9)	70	608.1 (481.1–768.7)	13	112.9 (65.6–194.5)

Abbreviations: CI, confidence interval; PY, person-years.

^a^Two cases, aged 4.5 and 11.5 years, were confirmed as Typhoid fever positive by a nonstudy private laboratory.

^b^One girl aged 4 years and one boy aged 8 years had coinfection with typhoid and paratyphoid.

**Table 4.** Clinical Features of Enteric Fevers

Clinical Features	Enteric Fever Episodes, No. (%)^a^		
	All Enteric Fever (n = 81)	Typhoid Fever (n = 70)	Paratyphoid Fever (n = 13)
Clinical symptoms (other than fever)			
Diarrhea/loose stools	14 (17.3)	13 (18.6)	2 (15.4)
Cough or cold	30 (37.0)	23 (32.9)	8 (61.5)
Abdominal pain	59 (72.8)	52 (74.3)	9 (69.2)
Nausea/vomiting	26 (32.1)	25 (35.7)	3 (23.1)
Headache	6 (7.4)	5 (7.1)	1 (7,7)
Not feeding well	14 (17.3)	13 (18.6)	1 (7.7)
Joint pain	1 (1.2)	1 (1.4)	0 (0.0)
Sore throat	3 (3.7)	2 (2.9)	1 (7.7)
Malaise	1 (1.2)	1 (1.4)	0
Duration of episode, mean (SD), d	9.7 (3.8)	10.1 (3.9)	7.7 (1.7)
Highest temperature during episode, mean (SD), ºC	38.9 (10.0)	38.9 (10.0)	39.2 (0.9)
Hospitalization during episode	12 (14.8)	12 (17.1)	1 (7.7)^b^
Duration of hospitalization, mean (SD), d	6.9 (2.8)	6.9 (2.8)	11
Outcome of enteric fever episodes			
Recovered	81 (98.8)	70 (98.6)	12 (92.3)
Recovered with complications^b^	1 (1.2)	1 (1.4)	1 (7.7)
Death	0 (0.0)	0 (0.0)	0 (0.0)

Abbreviation: SD, standard deviation.

^a^Data represent no. (%) of fever episodes unless otherwise specified.

^b^One patient who was coinfected with typhoid and paratyphoid had hemodynamic shock and recovered after 11 days of hospitalization. There were no reports of any other complications, including gastrointestinal bleeding or perforation, encephalopathy, myocarditis, hepatitis, or renal impairment.

## DISCUSSION

Our pediatric cohort showed a high burden of enteric fever, with typhoid more common than paratyphoid fever in low-income urban settings in Delhi. The incidence of both typhoid and paratyphoid was highest among children aged 10–15 years. Typhoid fever was seen to cause substantially prolonged illness compared with paratyphoid fever, with 17% of episodes requiring hospitalization. Typhoid and paratyphoid coinfections are not uncommon and can lead to prolonged illness and complications.

The global incidence of enteric fever is estimated as 197/100 000 PY in 2017, a 55% reduction from 1990 [12]. Despite a decline in the incidence of enteric fever over time, a substantial disease burden exists particularly in South Asia and sub-Saharan Africa. In South Asia, the estimated incidence of enteric fever in 2017 is 549/100 000 PY [12]. Because earlier estimates relied mostly on data extrapolation, various prospective facility-based studies in Asia and Africa (Surveillance for Enteric Fever in Asia Project and Severe Typhoid Fever Surveillance in Africa) and prospective cohorts with passive surveillance (Strategic Typhoid Alliance Across Africa and Asia) are ongoing, coupled with health utilization surveys [13]. Our SEFI (tier 1) study is a rigorous prospective population-based cohort with active surveillance. Among the tier 1 SEFI cohorts (Kolkata, Pune, Vellore, and Delhi) [9], Delhi was the only site where typhoid vaccine was introduced in the public health system in 2004 and population-based incidence estimates of typhoid fever were available before vaccine introduction.

The incidence of typhoid fever in our study was approximately 35% lower than in the 1995–1996 New Delhi cohort (980/100 000 PY) [2]. In the 1995–1996 Delhi cohort, the typhoid incidence was very high among children <5 years old (2730/100 000 PY) and showed a declining trend with increasing age. In our cohort, however, the typhoid incidence in children <5 years old was about 5 times lower, with an increasing trend with age. These differences may be due partly to contrasting methods but also to an actual reduction of disease burden over time. The 1995–1996 study was a dynamic cohort in which blood was collected for culture in children <5 years old irrespective of the duration of fever, while for participants >5 years old fever lasting ≥3 continuous days was required for blood culture. In our fixed cohort, the strategy to conduct blood culture among children with ≥3 consecutive days of fever was based on an initial exercise (as a part of all multicentric SEFI cohort sites) that showed low culture positivity (<1%) in 1- or 2-day fevers.

Other factors accountable to the typhoid burden reduction over time might be improvement in housing standards, WASH parameters and typhoid vaccine introduction among children <5 years old [3, 14–16]. During 1995–1996, the housing standards in South Delhi were poor, with 28% living in pucca concrete households [17], compared with >95% in the current study. Efforts to improve water supply systems and quality for all populations gained impetus with the Swachh Bharat Mission in 2014. In the 1995–1996 cohort, only about one-fourth of the households had access to a municipal piped water supply system [17], compared with 83% in the current cohort. In the 1990s, about 40% of the households in low-income urban neighborhoods in Delhi had drinking water contaminated with coliforms [18], compared with 15% in our current survey to generate population-based burden estimates in different geographic settings and provide a detailed understanding of the disease.

Similar to previous studies, enteric fever was more common during the monsoon and postmonsoon months, maybe because of the higher probability of water contamination during this period by mixing of drinking water sources with open sewers [19]. The higher number of enteric fever cases in 2019 might be because of higher rainfall in this year during the monsoon and postmonsoon seasons, compared with 2018 [20, 21]. However, we cannot rule out the possibility of higher case detection in the latter year, given the better experience of the team in following study procedures.

Our study findings have important implications. The high burden of typhoid fever, despite substantial improvements in water and sanitation practices, makes a stronger case for improving vaccination strategies to protect children both <5 years old and school-going children. The typhoid burden in our area exceeds the threshold of 300/100 000 PY, above which routine vaccination with TCV seems cost-effective [22]. The current vaccination strategy of a single dose of Vi polysaccharide vaccine at age 2 years in Delhi, without any subsequent dose, may explain the observed shift in disease toward older age group [23].

The epidemiological shift in disease has been documented in populations with low immunization coverage when specific age groups are vaccinated, classically exemplified by measles-mumps-rubella vaccine introduction among infants in Greece in 1975 [24, 25]. In our cohort, coverage of the polysaccharide vaccine was low (14%), possibly owing to lack of awareness and limited access to services in these low-income neighborhoods. These observations underscore the importance of considering the WHO recommendations on TCV vaccination in children >6 months of age and the need to consider strategies for catch-up vaccination and revaccination in older children [26], along with strengthening efforts to enhance population vaccine coverage.

Coinfection with *S*. Typhi and *S*. Paratyphi is not uncommon and has been reported elsewhere [27, 28]. These coinfections are difficult to treat and are often missed owing to indistinguishable clinical features. Given that the polysaccharide vaccine lacks ability to elicit cross-protection against paratyphoid, novel prevention strategies targeting both typhoid and paratyphoid together may be helpful. Improved point-of-care diagnostics are needed for quick and reliable diagnosis of enteric fever and early initiation of treatment and to minimize the unnecessary use of antityphoid antimicrobials.

Our study had several limitations. The incidence in our study seems to be an underestimate from the true disease burden because of (1) our strategy for blood culture, which precludes capture of enteric fevers with <3 consecutive days of febrile illness; (2) challenges to conduct blood culture tests in 21% of the eligible 3-day fever episodes (678 of 3234); (3) antibiotic use during the febrile episodes before blood culture; and (4) the limited blood culture sensitivity of about 60% for the diagnosis of enteric fever [29].

With the study culture positivity rate of about 3%, it is possible that about 20 of 678 of these missed eligible fever episodes may have tested positive in culture, giving an overall projected incidence rate of approximately 900/100 000 PY. Moreover, given that the median (IQR) duration of a fever episode is 2 (1–4) days, a biweekly contact would have allowed better capture of fever episodes at the cost of operational feasibility. We used a combination of active weekly contacts along with incentivization in form of monthly phone recharge for promoting passive reporting of fever. With our approach we may have missed some fever episodes, because many of these households have a common mobile phone, not always available for the primary caregiver of the child to report fever. A serosurvey measuring IgA or IgG may have provided alternative estimates of the rate of exposure to typhoid, but this was not operationally feasible within the scope of the tier 1 SEFI cohorts. Our study cohort is limited and underpowered to interpret the severity of disease. Finally, our study population is restricted to children <15 years old in a low-income urban slum area in Delhi, North India, and may not be generalizable to adults or other populations with different characteristics.

In conclusion, despite a 35% reduction in the overall incidence of typhoid fever compared with the 1995–1996 cohort, our study suggested a substantial burden of enteric fever among children in Delhi with higher burden among those aged 5–15 years. Moving forward, a holistic approach is needed to prevent the disease in all age groups. While the higher burden in the older children highlights the importance to discuss new vaccination strategies such as booster doses or catch-up immunization in school-going children >5 years of age, routine immunization with a single dose of TCV in infants aged >6 months, following WHO recommendations, could offer early and longer duration protection [26]. Continued efforts to improve vaccination coverage, accessibility of safe drinking water and sanitation facilities, education for hygienic health practices, and rigorous disease surveillance together can help achieve the goal of disease elimination.

## Supplementary Data

Supplementary materials are available at The Journal of Infectious Diseases online. Consisting of data provided by the authors to benefit the reader, the posted materials are not copyedited and are the sole responsibility of the authors, so questions or comments should be addressed to the corresponding author.

jiab046_suppl_Supplementary_Table_1

## Notes

***Acknowledgments.*** We thank Ankita Dutta, Ananya Tupaki-Sreepurna, and the whole team in the clinical and research laboratories at the Society for Applied Studies and the field team for the tier 1 Surveillance of Enteric Fever in India (SEFI) study at the Centre for Health Research and Development, Society for Applied Studies, for their intensive efforts. We are grateful for the guidance and support provided by Nita Bhandari, Senior scientist and Director at the Centre for Health Research and Development Society for Applied Studies, New Delhi. We acknowledge Duncan Steele, Megan Carey, and Supriya Kumar, Bill & Melinda Gates Foundation, and Nicholas Grassly, Imperial College London, for their technical support in proposal development. We also extend our thanks to Swathi Krishna and Arun S Kartikeyan from Christian Medical College, Vellore for their continued monitoring support as a part of the central quality assurance team.

***Study collaborators.*** SEFI tier 1 collaborators include Jacob John, Venkata Raghava Mohan, Ashish Bavdekar, Shanta Dutta, and Gagandeep Kang.

***Supplement sponsorship.*** This supplement is sponsored by the Christian Medical College Vellore Association.

***Financial support.*** This work is supported by the Bill & Melinda Gates Foundation (grant OPP1159351) through Christian Medical College Vellore.

***Potential conflicts of interest.*** All authors: No reported conflicts. All authors have submitted the ICMJE Form for Disclosure of Potential Conflicts of Interest. Conflicts that the editors consider relevant to the content of the manuscript have been disclosed.

## References

[1] John J , Van Aart CJ, Grassly NC. The burden of typhoid and paratyphoid in India: systematic review and meta-analysis. PLoS Negl Trop Dis 2016; 10:e0004616. 27082958 10.1371/journal.pntd.0004616 PMC4833325

[2] Sinha A , Sazawal S, Kumar R, et al. Typhoid fever in children aged less than 5 years. Lancet 1999; 354:734–7. 10475185 10.1016/S0140-6736(98)09001-1

[3] Sur D , Ochiai RL, Bhattacharya SK, et al. A cluster-randomized effectiveness trial of Vi typhoid vaccine in India. N Engl J Med 2009; 361:335–44. 19625715 10.1056/NEJMoa0807521

[4] Ochiai RL , Acosta CJ, Danovaro-Holliday MC, et al; Domi Typhoid Study Group. A study of typhoid fever in five Asian countries: disease burden and implications for controls. Bull World Health Organ 2008; 86:260–8. 18438514 10.2471/BLT.06.039818 PMC2647431

[5] Minutes of Meeting. State technical advisory group on immunization. Delhi, India: Directorate of Family Welfare, Government of NCT of Delhi, 2018. http://health.delhigovt.nic.in/wps/wcm/connect/5280968048f3e16dadc9bf26edbf4824/MAY2018MOMSTAGIMAIN.pdf?MOD=AJPERES&lmod=606309017&CACHEID=5280968048f3e16dadc9bf26edbf4824.

[6] Dewan D . Community-based typhoid vaccination program in New Delhi, India. Presented at: Eighth International Conference on Typhoid Fever and Other Invasive Salmonelloses; March 1-2, 2013.

[7] Mohan VK , Varanasi V, Singh A, et al. Safety and immunogenicity of a Vi polysaccharide-tetanus toxoid conjugate vaccine (Typbar-TCV) in healthy infants, children, and adults in typhoid endemic areas: a multicenter, 2-cohort, open-label, double-blind, randomized controlled phase 3 study. Clin Infect Dis 2015; 61:393–402. 25870324 10.1093/cid/civ295

[8] World Health Organization. Typhoid vaccines: WHO position paper, March 2018—recommendations. Vaccine 2019; 37:214–6. 29661581 10.1016/j.vaccine.2018.04.022

[9] John J , Bavdekar A, Rongsen-Chandola T, Dutta S, Kang G; NSSEFI Collaborators. Estimating the incidence of enteric fever in children in India: a multi-site, active fever surveillance of pediatric cohorts. BMC Public Health 2018; 18:594. 29724223 10.1186/s12889-018-5498-2 PMC5934828

[10] Balaji V , Kapil A, Shastri J, et al. Longitudinal typhoid fever trends in India from 2000 to 2015. Am J Trop Med Hyg 2018; 99:34–40. 30047367 10.4269/ajtmh.18-0139 PMC6128365

[11] Dutta A , More D, Tupaki-Sreepurna A, Sinha B, Goyal N, Rongsen-Chandola T. Typhoid and paratyphoid fever co-infection in children from an urban slum of Delhi. IDCases 2020; 20:e00717. 32195117 10.1016/j.idcr.2020.e00717 PMC7075973

[12] GBD 2017 Typhoid and Paratyphoid Collaborators. The global burden of typhoid and paratyphoid fevers: a systematic analysis for the Global Burden of Disease Study 2017. Lancet Infect Dis 2019; 19:369–81. 30792131 10.1016/S1473-3099(18)30685-6 PMC6437314

[13] Carey ME , MacWright WR, Im J, et al. The Surveillance for Enteric Fever in Asia Project (SEAP), Severe Typhoid Fever Surveillance in Africa (SETA), Surveillance of Enteric Fever in India (SEFI), and Strategic Typhoid Alliance Across Africa and Asia (STRATAA) population-based enteric fever studies: a review of methodological similarities and differences. Clin Infect Dis 2020; 71:102–10. 10.1093/cid/ciaa367 PMC7388711 32725221

[14] Prasad N , Jenkins AP, Naucukidi L, et al. Epidemiology and risk factors for typhoid fever in Central Division, Fiji, 2014–2017: a case-control study. PLOS Negl Trop Dis 2018; 12:e0006571. 29883448 10.1371/journal.pntd.0006571 PMC6010302

[15] Khan MI , Ochiai RL, Soofi SB, et al. Risk factors associated with typhoid fever in children aged 2-16 years in Karachi, Pakistan. Epidemiol Infect 2012; 140:665–72. 21676350 10.1017/S0950268811000938

[16] Sharma P , Taneja DK. Typhoid vaccine: a case for inclusion in national program. Indian J Public Health 2011; 55:267–71. 22298135 10.4103/0019-557X.92403

[17] Bahl R , Sinha A, Poulos C, et al. Costs of illness due to typhoid fever in an Indian urban slum community: implications for vaccination policy. J Health Popul Nutr 2004; 22:304–10. 15609783

[18] Bandyopadhyay S , Banerjee K, Khanna KK, Sharma RS, Verghese T. Drinking water quality and diarrhoea in Delhi. J Commun Dis 1992; 24:156–8. 1344946

[19] Saad NJ , Lynch VD, Antillón M, Yang C, Crump JA, Pitzer VE. Seasonal dynamics of typhoid and paratyphoid fever. Sci Rep 2018; 8:6870. 29720736 10.1038/s41598-018-25234-w PMC5932015

[20] Indian Meteorological Department. Annual climate summary 2019. Pune, India: National Climate Centre, Climate Services Division 2019.

[21] Indian Meteorological Department. Annual climate summary 2018. Pune, India: National Climate Centre, Climate Services Division 2018.

[22] Bilcke J , Antillón M, Pieters Z, et al. Cost-effectiveness of routine and campaign use of typhoid vi-conjugate vaccine in Gavi-eligible countries: a modelling study. Lancet Infect Dis 2019; 19:728–39. 31130329 10.1016/S1473-3099(18)30804-1 PMC6595249

[23] Pitzer VE , Bowles CC, Baker S, et al. Predicting the impact of vaccination on the transmission dynamics of typhoid in South Asia: a mathematical modeling study. PLoS Negl Trop Dis 2014; 8:e2642. 24416466 10.1371/journal.pntd.0002642 PMC3886927

[24] Gioula G , Fylaktou A, Exindari M, et al. Rubella immunity and vaccination coverage of the population of northern Greece in 2006. Euro Surveillance 2007; 12:E9-10. 10.2807/esm.12.11.00747-en 18005657

[25] Lahariya C . Vaccine epidemiology: a review. J Family Med Prim Care 2016; 5:7–15. 27453836 10.4103/2249-4863.184616 PMC4943153

[26] World Health Organization. Typhoid vaccines: WHO position paper—March 2018. Wkly Epi Rec 2018; 93:153–72. 10.1016/j.vaccine.2018.04.022 29661581

[27] Pratap CB , Kumar G, Patel SK, et al. Mix-infection of S. Typhi and ParaTyphi A in typhoid fever and chronic typhoid carriers: a nested PCR based study in North India. J Clin Diagn Res 2014; 8:Dc09-14. 10.7860/JCDR/2014/9167.5107 PMC4290235 25584217

[28] Debdas D , Joshi S. Mixed *Salmonella* infection. Indian J Med Microbiol 2008; 26:287. 18695342 10.4103/0255-0857.42073

[29] Voysey M , Pant D, Shakya M, et al. Under-detection of blood culture-positive enteric fever cases: the impact of missing data and methods for adjusting incidence estimates. PLoS Negl Trop Dis 2020; 14:e0007805. 31945052 10.1371/journal.pntd.0007805 PMC6964825

