# Supplementary material for: Incidence of Enteric Fever in a Pediatric Cohort in North India: Comparison with Estimates from 20 Years Earlier
Source: J Infect Dis. 2021 Nov 23;224(Suppl 5):S558–67. doi: 10.1093/infdis/jiab046 (PMC8892529; doi:10.1093/infdis/jiab046)
Supplement: jiab046_suppl_Supplementary_Table_1 [file jiab046_suppl_supplementary_table_1.docx]

**Supplementary Table1. Antibiotic Susceptibility of the isolated enteric fever strains**

| **Antibiotics** | **Typhoid (n=68)** | | | **Paratyphoid (n=13*)** | | |
| --- | --- | --- | --- | --- | --- | --- |
|  | **S** | **I** | **R** | **S** | **I** | **R** |
| **Ampicillin** | 68 | 0 | 0 | 13 | 0 | 0 |
| **Azithromycin** | 68 | 0 | 0 | 13 | 0 | 0 |
| **Ceftriaxone** | 68 | 0 | 0 | 13 | 0 | 0 |
| **Chloramphenicol** | 67 | 0 | 1 | 13 | 0 | 0 |
| **Ciprofloxacin** | 1 | 50 | 17 | 0 | 13 | 0 |
| **Cotrimoxazole** | 67 | 0 | 1 | 13 | 0 | 0 |
| **Pefloxacin** | 10 | 0 | 58 | 1 | 0 | 12 |
| **S= Sensitive; I= Intermediate; R= Resistant as per CLSI standards for Salmonella**  *2 cases have co-infection with typhoid and paratyphoid  AST available for 79 positive cases since 2 culture are the outside | | | | | | |
